# Supplementary material for: A versatile information retrieval framework for evaluating profile strength and similarity
Source: Nat Commun. 2025 Jun 4;16:5181. doi: 10.1038/s41467-025-60306-2 (PMC12137819; doi:10.1038/s41467-025-60306-2)
Supplement: Supplementary file 2 — Reporting Summary [file 41467_2025_60306_MOESM2_ESM.pdf]

nature portfolio

Corresponding author(s): Gregory P. Way, gregory.way@cuanschutz.edu, Shantanu Singh, shantanu@broadinstitute.org

Last updated by author(s): Apr 16, 2025

Reporting Summary

Nature Portfolio wishes to improve the reproducibility of the work that we publish. This form provides structure for consistency and transparency in reporting. For further information on Nature Portfolio policies, see our Editorial Policies and the Editorial Policy Checklist.

Statistics

For all statistical analyses, confirm that the following items are present in the figure legend, table legend, main text, or Methods section.

n/a	Confirmed	
☐	☒	The exact sample size (*n*) for each experimental group/condition, given as a discrete number and unit of measurement
☐	☒	A statement on whether measurements were taken from distinct samples or whether the same sample was measured repeatedly
☐	☒	The statistical test(s) used AND whether they are one- or two-sided<br>*Only common tests should be described solely by name; describe more complex techniques in the Methods section.*
☐	☒	A description of all covariates tested
☐	☒	A description of any assumptions or corrections, such as tests of normality and adjustment for multiple comparisons
☐	☒	A full description of the statistical parameters including central tendency (e.g. means) or other basic estimates (e.g. regression coefficient) AND variation (e.g. standard deviation) or associated estimates of uncertainty (e.g. confidence intervals)
☐	☒	For null hypothesis testing, the test statistic (e.g. *F*, *t*, *r*) with confidence intervals, effect sizes, degrees of freedom and *P* value noted<br>*Give P values as exact values whenever suitable.*
☒	☐	For Bayesian analysis, information on the choice of priors and Markov chain Monte Carlo settings
☒	☐	For hierarchical and complex designs, identification of the appropriate level for tests and full reporting of outcomes
☒	☐	Estimates of effect sizes (e.g. Cohen's *d*, Pearson's *r*), indicating how they were calculated

Our web collection on statistics for biologists contains articles on many of the points above.

Software and code

Policy information about availability of computer code

Data collection	No software was used
Data analysis	The mAP framework implementation is available as an open-source Python package: https://github.com/cytomining/copairs

For manuscripts utilizing custom algorithms or software that are central to the research but not yet described in published literature, software must be made available to editors and reviewers. We strongly encourage code deposition in a community repository (e.g. GitHub). See the Nature Portfolio guidelines for submitting code & software for further information.

Data

Policy information about availability of data

All manuscripts must include a data availability statement. This statement should provide the following information, where applicable:

- Accession codes, unique identifiers, or web links for publicly available datasets
- A description of any restrictions on data availability
- For clinical datasets or third party data, please ensure that the statement adheres to our policy

Profiles extracted from the Cell Health dataset are available at https://github.com/broadinstitute/cell-health/tree/30ea5de393eb9cfc10b575582aa9f0f857b44c59/1.generate-profiles. Profiles extracted from the cpg0004 dataset are available at https://github.com/broadinstitute/lincs-cell-painting/tree/061870127481dcd73c29df85ebcfddeac2ed0828/profiles. Profiles extracted from the cpg0016[orf] dataset are available from the Cell Painting Gallery at https://github.com/broadinstitute/cellpainting-gallery/blob/87e04696564e8c61d060c2a8e3a99dbd00fd9b31/README.md. The Perturb-seq dataset is available at Gene Expression Omnibus, accession code GSE13208061 [https://www.ncbi.nlm.nih.gov/geo/query/acc.cgi?acc=GSE132080]. Profiles

extracted from the matching nELISA-Cell Painting dataset are available at https://github.com/carpenter-singh-lab/2024_Kalinin_mAP/tree/e9a5414726119dca7ed0d79efde887c1e259c288/experiments/5_nelisa/inputs. Profiles extracted from the Mitocheck dataset are available at https://github.com/WayScience/mitocheck_data/blob/613acbb20d2134ad1d725c7605a61c5a9e823c1a/README.md.

Research involving human participants, their data, or biological material

Policy information about studies with human participants or human data. See also policy information about sex, gender (identity/presentation), and sexual orientation and race, ethnicity and racism.

Reporting on sex and gender	N/A
Reporting on race, ethnicity, or other socially relevant groupings	N/A
Population characteristics	N/A
Recruitment	N/A
Ethics oversight	N/A

Note that full information on the approval of the study protocol must also be provided in the manuscript.

Field-specific reporting

Please select the one below that is the best fit for your research. If you are not sure, read the appropriate sections before making your selection.

[x] Life sciences [ ] Behavioural & social sciences [ ] Ecological, evolutionary & environmental sciences

For a reference copy of the document with all sections, see nature.com/documents/nr-reporting-summary-flat.pdf

Life sciences study design

All studies must disclose on these points even when the disclosure is negative.

Sample size	No data collection was performed in this study. No statistical methods were used to predetermine the sample size. We analyzed existing datasets created in previous studies.
Data exclusions	As part of the data preprocessing, we filtered certain columns of the data matrices (feature selection) as described in Methods.
Replication	We did not perform biological or technical replication in this study as all data was created in the previous studies.
Randomization	Computing a null distribution for mAP involved randomly shuffling perturbation labels. No other randomization was performed.
Blinding	There was no blinding performed in this study because the samples were not evaluated by human experts.

Reporting for specific materials, systems and methods

We require information from authors about some types of materials, experimental systems and methods used in many studies. Here, indicate whether each material, system or method listed is relevant to your study. If you are not sure if a list item applies to your research, read the appropriate section before selecting a response.

Materials & experimental systems

n/a	Involved in the study	
x		Antibodies
x		Eukaryotic cell lines
x		Palaeontology and archaeology
x		Animals and other organisms
x		Clinical data
x		Dual use research of concern
x		Plants

Methods

n/a	Involved in the study	
x		ChIP-seq
x		Flow cytometry
x		MRI-based neuroimaging

Plants

Seed stocks	N/A
Novel plant genotypes	N/A
Authentication	N/A
